# CircRNA Profiling of Skeletal Muscle in Two Pig Breeds Reveals CircIGF1R Regulates Myoblast Differentiation via miR-16

**DOI:** 10.3390/ijms24043779

**Published:** 2023-02-14

**Authors:** Meng Li, Na Zhang, Jiao Li, Mengting Ji, Tianzhi Zhao, Jiaqi An, Chunbo Cai, Yang Yang, Pengfei Gao, Guoqing Cao, Xiaohong Guo, Bugao Li

**Affiliations:** College of Animal Science, Shanxi Agricultural University, Taigu, Jinzhong 030801, China

**Keywords:** pig, skeletal muscle, circIGF1R, miR-16, myoblast differentiation

## Abstract

Muscle development is closely related to meat quality and production. CircRNAs, with a closed-ring structure, have been identified as a key regulator of muscle development. However, the roles and mechanisms of circRNAs in myogenesis are largely unknown. Hence, in order to unravel the functions of circRNAs in myogenesis, the present study explored circRNA profiling in skeletal muscle between Mashen and Large White pigs. The results showed that a total of 362 circRNAs, which included circIGF1R, were differentially expressed between the two pig breeds. Functional assays showed that circIGF1R promoted myoblast differentiation of porcine skeletal muscle satellite cells (SMSCs), while it had no effect on cell proliferation. In consideration of circRNA acting as a miRNA sponge, dual-luciferase reporter and RIP assays were performed and the results showed that circIGF1R could bind miR-16. Furthermore, the rescue experiments showed that circIGF1R could counteract the inhibitory effect of miR-16 on cell myoblast differentiation. Thus, circIGF1R may regulate myogenesis by acting as a miR-16 sponge. In conclusion, this study successfully screened candidate circRNAs involved in the regulation of porcine myogenesis and demonstrated that circIGF1R promotes myoblast differentiation via miR-16, which lays a theoretical foundation for understanding the role and mechanism of circRNAs in regulating porcine myoblast differentiation.

## 1. Introduction

Skeletal muscle is the main source of high-quality protein for human beings, which is closely related to animal economic traits. Skeletal muscle development is achieved through the coordinated and orderly expression of transcription factors. In addition to transcription factors, miRNAs, lncRNAs, and circRNAs have been identified in recent years, which play essential roles in skeletal muscle development [1,2]. CircRNAs are closed-loop and are formed by reverse splicing [3]. Compared with other linear RNAs, circRNAs have higher stability and RNase R resistance [4,5]. Thus, circRNAs are considered ideal biomarkers. Owing to these characteristics, circRNAs have been widely used in the diagnosis, treatment, and drug development of numerous diseases [6,7]. At present, circRNAs have been successfully used in the research and development of COVID-19 vaccines [8]. However, there are few pieces of evidence about the circRNAs’ function in muscle development. Therefore, the identification of novel functional circRNAs involved in myogenesis and elucidation of their mechanisms require further investigation.

To date, circRNAs have been detected in many species [9,10,11]; they are plentiful in skeletal muscle [12,13] and are key regulators of skeletal muscle development [14,15,16]. CircRNAs regulate myoblast differentiation through a ceRNA mechanism. For example, it is known that circFGFR2 promotes myogenesis via miR-133a-5p [17], and Ru et al. found that circCPE competitively binds miR-138 to inhibit myoblast differentiation in cattle [18]. It is thus evident that circRNAs have an important function during muscle development, but the roles and mechanisms of circRNAs in myogenesis are largely unknown.

The Mashen pig is a nationally protected breed in China, which has a higher meat quality and slower growth rate than the Large White pig. There were significant differences in muscle fiber diameter, muscle fiber composition, and intramuscular fat (IMF) content between Mashen and Large White pigs [19]. Mashen pigs have higher muscle density, IMF content, and expression levels of MyHCI and MyHCIIa. Therefore, the two pig breeds are ideal models for studying muscle development. Based on these phenotypic differences, RNA sequencing was performed on skeletal muscles at three developmental stages in two pig breeds to screen candidate circRNAs regulating muscle development, which is meaningful for understanding the mechanism of muscle development and improving meat quality.

The aim of this study was as follows: to screen the circRNAs that regulate myogenesis in pigs and to explore the regulatory effects and mechanisms of circRNAs on myogenic differentiation of porcine SMSCs. In this respect, RNA sequencing of the *longissimus dorsi* in Large White and Mashen pigs was conducted to screen out differentially expressed circRNAs (DEcircRNAs). If DEcircRNAs can be screened, these circRNAs may be involved in regulating porcine muscle development. Western blot, immunofluorescence, dual-luciferase reporter, and RIP assays were then employed to detect the effect and mechanism of DEcircRNAs on the myogenic differentiation of porcine SMSCs. Our results provide a foundation for conducting in-depth analyses of the role of circRNAs in myogenesis, which will be meaningful for genetically improving pork yield and quality.

## 2. Results

### 2.1. DEcircRNAs were Analyzed between Mashen and Large White Pigs

**Figure 1:**
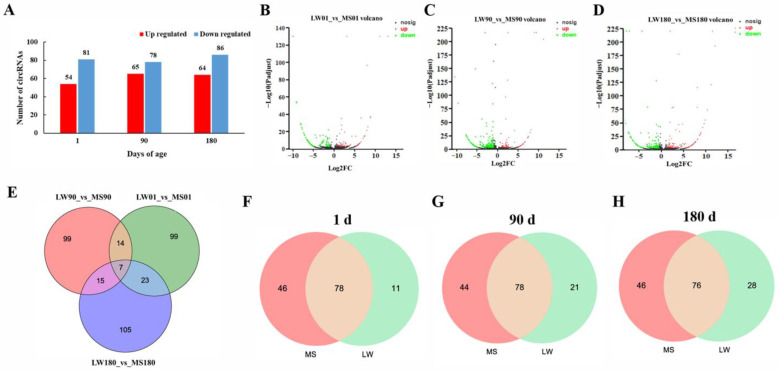
DEcircRNA analysis between two breeds. (**A**): Numner of DEcircRNAs. (**B**–**D**): Volcano plot of DEcircRNAs at 1, 90, and 180 days of age, respectively. (**E**): Venn diagram of DEcircRNAs. (**F**–**H**): Venn diagram showing the expression specificity of DEcircRNAs between the two pig species.

To screen for candidate circRNAs that regulate pig muscle development, RNA sequencing was performed on the *longissimus dorsi* of Mashen and Large White pigs. A total of 362 DEcircRNAs were screened between the two pig breeds by conducting a differentially expressed circRNA analysis (Figure 1A). Specifically, there were 54 up-regulated DEcircRNAs and 81 down-regulated DEcircRNAs in Mashen pigs at 1 day of age, 65 up-regulated DEcircRNAs and 78 down-regulated DEcircRNAs in Mashen pigs at 90 days of age, and 64 up-regulated DEcircRNAs and 86 down-regulated DEcircRNAs in Mashen pigs at 180 days of age. Figure 1B–D shows a volcano plot of the DEcircRNAs. A further analysis of DEcircRNAs showed that seven DEcircRNAs were different at all three stages between two pig breeds. Twenty-one DEcircRNAs were differently expressed at 1 and 90 days of age, 30 were different expressed at 1 and 180 days of age, and 22 were significantly different expressed at 90 and 180 days (Figure 1E). Figure 1F–H shows the expression specificity of DEcircRNAs between the two pig breeds. At 1 day of age, 46 and 11 DEcircRNAs were found to be specifically expressed in Mashen and Large White pigs, respectively. The results determined at the ages of 90 and 180 days are shown in Figure 1G,H. A heatmap depicting the expression profiles of DEcircRNAs is provided in Figure S2.

**Figure 2:**
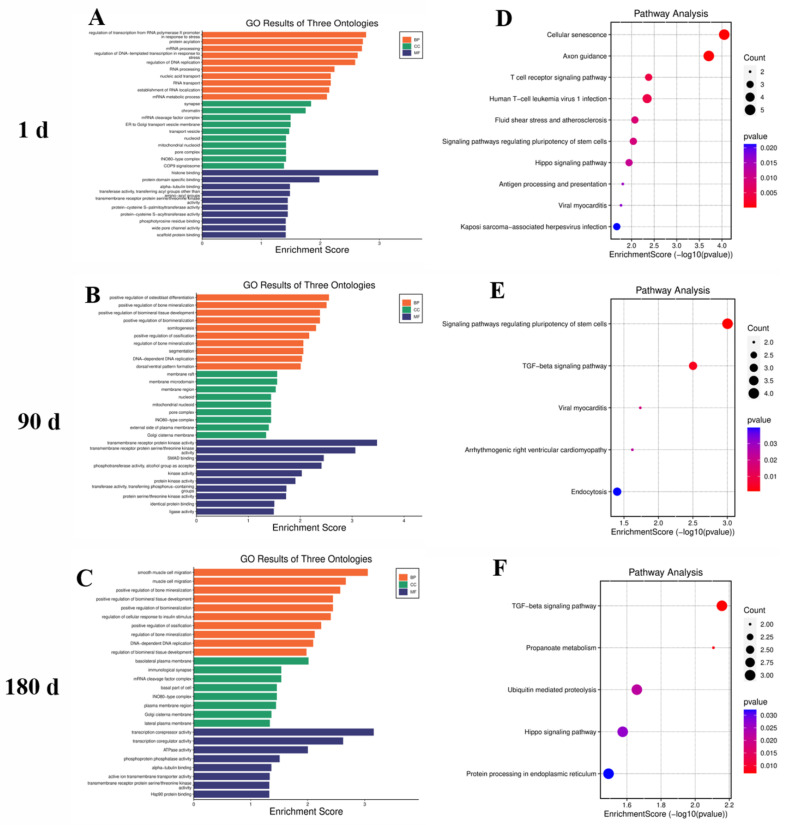
Functional enrichment results of the host genes of DEcircRNAs. (**A**,**D**): GO and KEGG analysis at 1 day of age; (**B**,**E**): 90 days of age; and (**C**,**F**): 180 days of age.

An enrichment analysis showed that the host genes of DEcircRNAs at 1 day of age were mainly enriched in cellular senescence, the Hippo signaling pathway, and antigen processing and presentation (Figure 2A,D); those at 90 days of age were mainly enriched in the TGF-beta signaling pathway and endocytosis (Figure 2B,E); and those at 180 days of age were mainly enriched in propanoate metabolism and the TGF-beta and Hippo signaling pathways (Figure 2B,E).

### 2.2. Construction of the ceRNA Network Regulating Porcine Muscle Development

**Figure 3:**
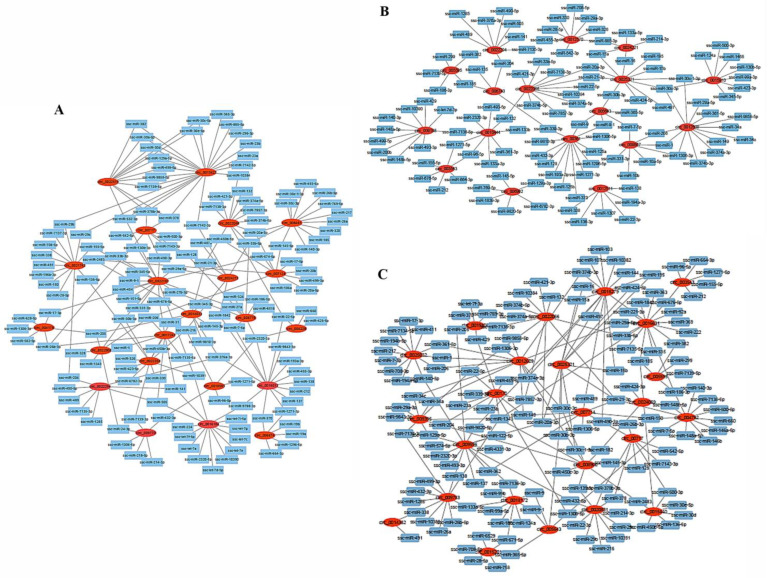
CeRNA network of DEcircRNAs. (**A**): 1 day of age; (**B**): 90 days of age; and (**C**): 180 days of age.

The ceRNA network of DEcircRNAs and target miRNAs was constructed at 1, 90, and 180 days of age. The target miRNAs of DEcircRNAs at 1 day of age are shown in Figure 3A. Specifically, circ_0010581, circ_0013627, circ_0014033, circ_0016012, circ_0016104, circ_0017247, circ_0021742, circ_0022187, circ_0022294, circ_0022358, circ_0022519, circ_0022905, circ_0024069, circ_0024215, circ_004228, circ_006674, circ_007124, circ_00717, circ_008481, circ_008778, circ_009770, and circ_0022066 had 5, 28, 8, 14, 8, 13, 18, 8, 11, 12, 7, 3, 7, 10, 6, 11, 8, 13, 8, 6, and 11 target miRNAs, respectively. These miRNAs included ssc-let-7, ssc-miR-19, and ssc-miR-20, which may be involved in the regulation of muscle development. The specific target miRNAs of DEcircRNAs at 90 days of age are shown in Figure 3B, and these include ssc-miR-15, ssc-miR-16, and ssc-miR-133 (Figure 3B). Detailed information about the target miRNAs of DEcircRNAs at 180 days of age are shown in Figure 3B, and these include ssc-miR-16, ssc-miR-17, and ssc-miR-133 (Figure 3C).

### 2.3. CircIGF1R Promotes Myoblast Differentiation of Porcine SMSCs

CircIGF1R was found to be highly expressed among DEcircRNAs (mean fpkm value: 518). We also found that circIGF1R was not only differentially expressed between the two pig breeds, but also in the porcine *longissimus dorsi* at different developmental stages. Besides, the circIGF1R-derived gene has an important function in muscle development. For the above reasons, circIGF1R was selected for further functional studies.

**Figure 4:**
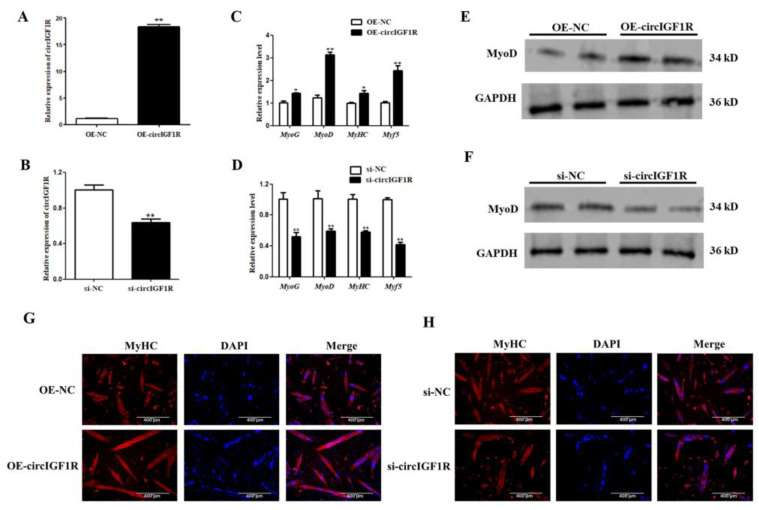
Effect of circIGF1R on myoblast differentiation of porcine SMSCs. (**A**,**B**): Cell transfection efficiency. (**C**,**D**): Myogenic factor expression at the mRNA level. (**E**,**F**): MyoD protein expression. (**G**,**H**): Cell immunofluorescence. Blue indicates nuclei stained with DAPI; red indicates MyHC protein. ** *p* < 0.01, * *p* < 0.05.

CircIGF1R was overexpressed/interfered in porcine SMSCs. CircIGF1R expression was significantly higher in the OE-circIGF1R group (*p* < 0.01; Figure 4A) and was significantly decreased in the interference group (*p* < 0.01, Figure 4B). 

After overexpression/interference of circIGF1R, porcine SMSCs were induced to differentiate, and the cells were collected after myoblast differentiation for 4 days. The mRNA levels of MyoD, MyoG, MyHC, and Myf5 were significantly increased after circIGF1R overexpression (*p* < 0.05, Figure 4C). Western blot results showed that MyoD protein expression was also significantly increased after circIGF1R overexpression (Figure 4E). Immunofluorescence results showed that the number of myoducts was higher and the myotube was thicker in the overexpression group (Figure 4G). The opposite results were obtained with circIGF1R interference (Figure 4D,F,H). These results indicate that circIGF1R promotes myoblast differentiation in porcine SMSCs.

### 2.4. CircIGF1R Has no Effect on the Proliferation of Porcine SMSCs 

**Figure 5:**
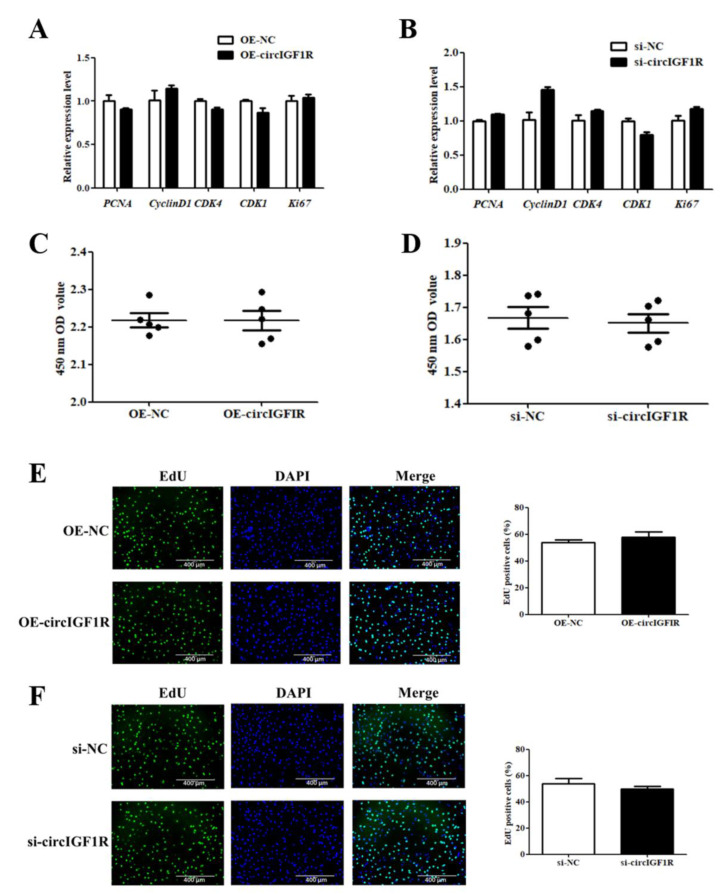
Effect of circIGF1R on the proliferation of porcine SMSCs. (**A**,**B**): Expression level changes of genes related to cell proliferation. (**C**,**D**): The results of CCK8. (**E**,**F**): The results of EdU.

When circIGF1R was overexpressed/interfered in porcine SMSCs, there was no difference in proliferation-related genes expression (*p* > 0.05, Figure 5A,B). CCK8 results showed that there was no significant difference in the number of cells at the proliferation stage in the treatment group (*p* > 0.05, Figure 5C,D), and the same results were obtained with EdU (*p* > 0.05, Figure 5E,F). These results indicated that circIGF1R has no effect on the proliferation of porcine SMSCs.

### 2.5. CircIGF1R Serves as a miR-16 Sponge

**Figure 6:**
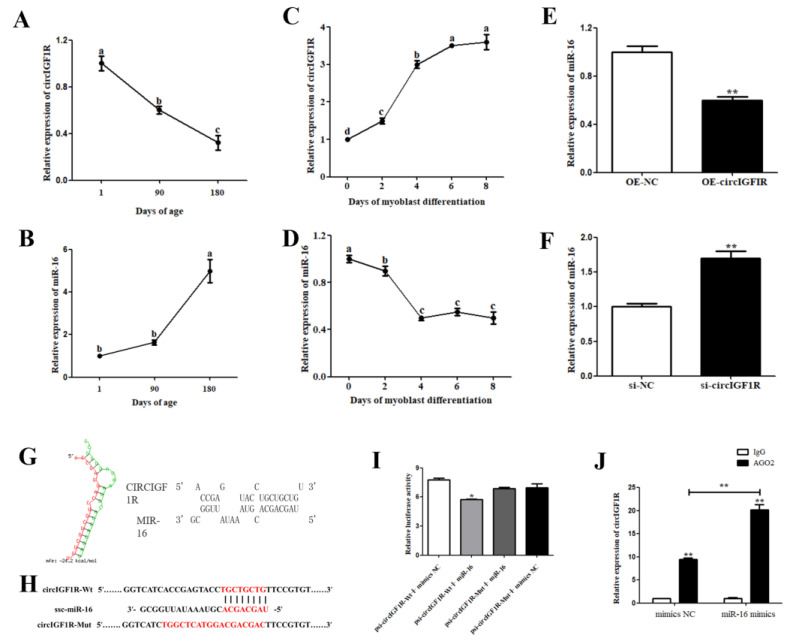
CircIGF1R serves as a miR-16 sponge. (**A**,**B**): circIGF1R and miR-16 expression in muscle tissue at different stages. (**C**,**D**): circIGF1R and miR-16 expression during the myoblast differentiation of pig satellite cells. (**E**,**F**): miR-16 expression after overexpression/interference of circIGF1R. (**G**): RNhybrid was used to predict binding sites of circIGF1R and miR-16. (**H**): The sequence of wild and mutant type vectors. (**I**): Dual-luciferase reporter assay. (**J**): AGO2-RIP assay. Different lower case letters indicate *p* < 0.05. ** *p* < 0.01, * *p* < 0.05.

The prediction of circIGF1R-bound miRNAs revealed that circIGF1R had multiple miRNA targets. Of these, miR-16 had the strongest negative correlation with circIGF1R (Figure S1). Therefore, we further explored the mechanism of circIGF1R regulating myogenesis through miR-16. CircIGF1R showed an upward trend with an increase in the day age of pig skeletal muscle (Figure 6A) and during the myoblast differentiation of satellite cells (Figure 6C). miR-16 showed a downward trend with increasing day age and differentiation days (Figure 6B,D); the expression trend of miR-16 was thus opposite to that of circIGF1R. In addition, the overexpression of circIGF1R in porcine SMSCs significantly reduced miR-16 expression (*p* < 0.01, Figure 6E), while the interference of circIGF1R increased miR-16 expression (*p* < 0.01, Figure 6F). These results show that miR-16 may be negatively regulated by circIGF1R.

RNhybrid online software predicted that miR-16 forms a secondary structure with circIGF1R through base complementary pairing with a minimum binding free energy of −24.2 kcal/mol, which is evidence of a strong binding ability (Figure 6G). The psiCHECK2-circIGF1R-Wt and psiCHECK2-circIGF1R-Mut vectors are shown in Figure 6H. The dual-luciferase reporter demonstrated that luciferase activity was reduced when co-transfected with psiCHECK2-circIGF1R-Wt+miR-16 mimics compared with that after transfection with psiCHECK2-circIGF1R-Mut+miR-16 mimics. Compared with the co-transfection of psiCHECK2-circIGF1R-Wt+mimics NC, luciferin activity was also significantly decreased. A binding effect between circIGF1R and miR-16 was observed (*p* < 0.05, Figure 6I).

The RIP results showed that the AGO2 antibody group enriched greater amounts of miR-16 and circIGF1R, which indicated that both miR-16 and circIGF1R could bind to AGO2, thus possibly playing a role through a ceRNA mechanism. After transfection with miR-16 mimics, the miR-16 expression of the AGO2 antibody group was increased (*p* < 0.01, Figure 6J). Moreover, circIGF1R enrichment by the AGO2 antibody in the miR-16 mimics-transfected group was higher than that of the NC group (*p* < 0.01, Figure 6J). These results further indicate that circIGF1R serves as an miR-16 sponge.

### 2.6. CircIGF1R Promotes Myoblast Differentiation of SMSCs via miR-16

**Figure 7:**
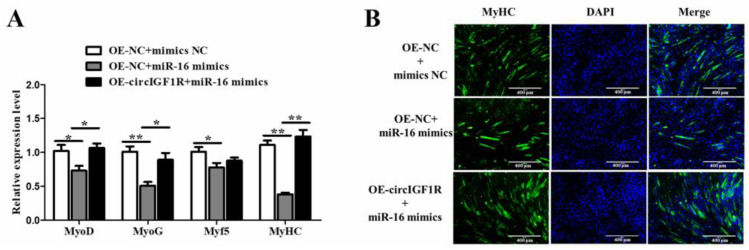
CircIGF1R promotes myoblast differentiation of SMSCs via miR-16. (**A**): Expression changes of key myogenic factors after transfection with OE-NC+mimics NC, OE-NC+miR-16 mimics, and OE-circIGF1R+miR-16 mimics. (**B**): Immunofluorescence staining results after transfection with OE-NC+mimics NC, OE-NC+miR-16 mimics, and OE-circIGF1R+miR-16 mimics. Blue indicates nuclei stained with DAPI; green indicates MyHC protein. ** *p* < 0.01, * *p* < 0.05.

Rescue experiments demonstrated a significant decrease in the myogenic factor expression of the co-transfected OE-NC+miR-16 mimics group (*p* < 0.05, Figure 7A) and the myotube fusion index (Figure 7B), which proves that miR-16 inhibits myogenic differentiation of porcine SMSCs. The function of miR-16 is, therefore, opposite to that of circIGF1R. In addition, compared with the co-transfected OE-NC+miR-16 mimics group, the expression of muscle factors in the OE-circIGF1R+miR-16 group was significantly increased (*p* < 0.05, Figure 7A) together with the myotube fusion index (Figure 7B). In conclusion, circIGF1R alleviates the inhibiting effect of miR-16 on the myogenesis of porcine SMSCs.

### 2.7. Translation Ability Prediction of CircIGF1R

**Figure 8:**
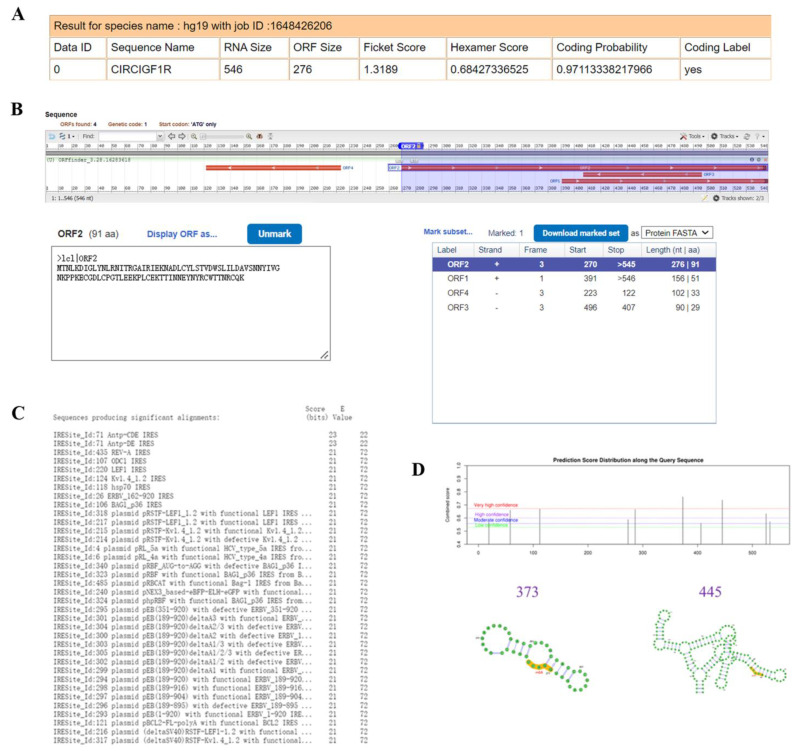
Prediction of the translation ability of circIGF1R. (**A**,**B**): Translation ability and ORF prediction of circIGF1R. (**C**): IRES prediction of circIGF1R. (**D**): m6A site prediction of circIGF1R.

In addition to the ceRNA mechanism, circIGF1R may also function via translation into protein. It has been found that some circRNAs translate into proteins/peptides through IRES or m6A-mediated translation initiation. The CPAT (Figure 8A) and ORF Finder (Figure 8B) online websites predicted that circIGF1R had open reading box. Of these, 270 bp to 545 bp were public ORFs on the two websites, and these encode for 92 amino acids. The CPAT website predicted that circIGF1R had a coding probability of 97.1%. Furthermore, the IRESite online website predicted that circIGF1R had multiple IRES (Figure 8C), and the SRAMP online website found a total of 10 m6A sites in circIGF1R (Figure 8D), with high binding sites at 373 bp and 445 bp (Figure 8D). The above indicate that circIGF1R has ORF, IRES, and m6A sites, which may be translated into small peptides. Our research group is currently studying the function of the putative protein of circIGF1R.

## 3. Discussion

The present study screened a total of 362 DEcircRNAs, which regulate skeletal muscle development, between Mashen and Large White pigs. This study provides the first ever proof that circIGF1R promotes myoblast differentiation of porcine SMSCs via serving as a miR-16 sponge. Our results are meaningful for improving pork yield and quality.

CircIGF1R is an exonic circRNA generated by the second exon of the *IGF1R* gene, and it is highly expressed in the muscle and fat tissues of pigs [20,21]. As a member of the insulin-like growth factor system (IGFs), IGF1R regulates cell proliferation, the cell cycle, and autophagy, mainly by activating multiple signaling pathways. IGF1R is an essential regulatory receptor for normal cell growth [22,23,24]. It is also known that loss of IGF1R specifically in muscle leads to a mild decrease in muscle size [25,26], and insulin and IGF1R receptors regulate FoxO-mediated signaling in muscle proteostasis [27]. As IGF1R participates in muscle development, the function of circIGF1R in regulating muscle development requires further study.

CircIGF1R has been identified in humans and mice, and has been demonstrated to have a variety of biological functions. Previous studies have shown that silencing CircIGF1R protects against neuronal injury [28], regulates psoriasis via miR-194-5p/CDK1 [29], and inhibits cell invasion and migration in lung cancer [30]. In addition, circRNAs are known to have tissue and developmental stage expression specificity and multiple biological regulatory functions [31,32]. Our previous study showed that circIGF1R promotes lipogenic differentiation of porcine preadipocytes [21], but the current study is the first to demonstrate that circIGF1R can promote myogenic differentiation of SMSCs.

**Figure 9:**
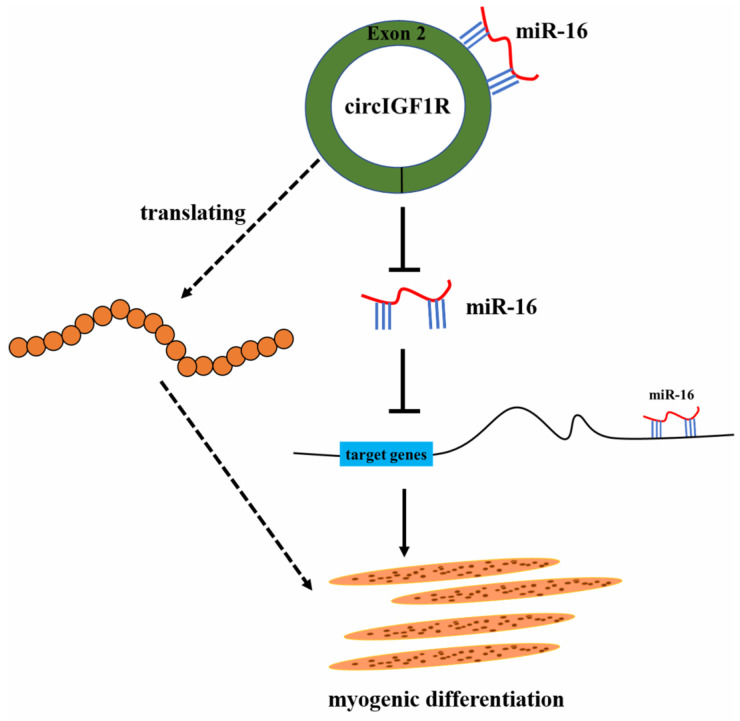
Mechanism of circIGF1R regulating myoblast differentiation through miR-16.

CircRNAs regulate myoblast differentiation through a ceRNA mechanism. In the present study, we demonstrated that circIGF1R promotes myoblast differentiation of porcine SMSCs through competitive binding of miR-16. Functional research on miR-16 has mainly focused on diseases [33,34,35,36]. Muscle miR-16 deletion results in impaired insulin sensitivity and contractile function in a sex-dependent manner [37]. miR-16-5p suppression protects human cardiomyocytes against endoplasmic reticulum and oxidative stress-induced injury [38]. However, few studies have investigated the regulation of muscle development by miR-16. In this study, miR-16 was proven to inhibit myoblast differentiation of SMSCs, in contrast to the regulation of myoblast differentiation by circIGF1R. In addition, circIGF1R relieved the inhibitory effect of miR-16 on SMSCs myogenesis. Therefore, our results prove that circIGF1R promotes myoblast differentiation of SMSCs via miR-16 (Figure 9).

## 4. Materials and Methods

### 4.1. Muscle Samples Collection

Experimental protocols were reviewed and approved by the Animal Ethics Committee of Shanxi Agricultural University (Shanxi, China, SXAU-EAW-P002003). In the present study, a total of nine healthy Mashen and nine healthy Large White male pigs were selected from the Datong Pig Breeding Farm (Taigu, China). Three pigs each from both breeds were slaughtered at 1 (early stage), 90 (middle stage), and 180 (later stage) days after birth. The longissimus dorsi was collected, snap-frozen in liquid nitrogen, and stored at −80 °C for further use. Longissimus dorsi samples were used for RNA sequencing. Specific information is provided in a previously published article [20].

### 4.2. Sequencing Data Analysis

The sequencing method and bioinformatic analysis method followed those presented in a previous study [20]. Total RNA was isolated from muscle tissue samples using TRIzol^®^ Reagent (Invitrogen, Carlsbad, CA, USA). Ribosomal RNAs (rRNAs) were removed from total RNA using a Ribo-Zero Magnetic kit (Epicentre, Madison, WI, USA), and the remaining RNA obtained after rRNA removal was fragmented to approximately 200 bp. A TruSeqTM Stranded Total RNA Library Prep Kit (Illumina, San Diego, CA, USA) was used to prepare the cDNA library. Fragmented RNAs were employed to generate first-strand cDNA using random primers. For second-strand synthesis, dTTP was substituted with dUTP in the dNTP reagent, thereby allowing the second base of the cDNA chain to contain A/U/C/G. Following end-repair and A-tailing, 150–200 bp cDNA fragments were isolated, and double-stranded cDNA was ligated to a “Y” adaptor. Single-strand cDNA was then obtained using uracil-N-glycosylase (UNG). Next, PCR amplification was performed to enrich the cDNA libraries. Finally, paired-end sequencing was conducted in the present study, and sequencing was performed on an Illumina HiSeq sequencing platform. The sequencing data were then subjected to quality control. CIRI software was selected to identify circRNAs for subsequent analysis. The expression level of circRNA was estimated via the number of back-spliced reads. In this study, the spliced reads per billion mapping method was used to estimate the circRNA expression level. Furthermore, edgeR was used to analyze DEcircRNAs (FDR < 0.05 and |log2 Fold change (FC)| ≥ 1). GO and KEGG analyses of DEcircRNAs’ host genes were conducted. TargetFinder and RNAhybrid software packages were used to predict the target miRNAs of DEcircRNAs and the binding sites between DEcircRNAs and miRNAs. Finally, cytoscape software was used to construct the DEcircRNAs-miRNAs regulatory network.

### 4.3. Cell Culture and Transfection

Newborn piglets were sacrificed by bloodletting, the whole body was cleaned with 75% alcohol, and the longissimus dorsi muscle was excised. Collagenase I (Sigma, Saint Louis, MO, USA) was used to digest muscle tissue at 37 °C for 1 h. The satellite cells were purified by differential adhesion. Purified cells were cultured in DMEM containing 20% FBS (Gibco, Grand Island, NY, USA). When the cells had grown to 70–80% confluence, a differentiation medium (DMEM containing 2% horse serum) was used to induce myoblast differentiation. Lipofectamine 3000 (Thermo Fisher Scientific, Waltham, MA, USA) was employed to transfect the cells.

### 4.4. qRT-PCR

Total RNA was isolated using TRIzol reagent (Takara, Shiga, Japan). cDNA was synthesized using the PrimeScript RT reagent Kit with gDNA Eraser (Takara, Japan) and the miRNA 1st Strand cDNA Synthesis Kit (by stem-loop) (Vazyme, Nanjing, China). qRT-PCR was performed using the SYBR PrimeScriptTM RT-PCR Kit (Takara, Japan) and the miRNA Universal SYBR qPCR Master Mix (Vazyme, Nanjing, China). All primers used are listed in Tables S1 and S2. The quantitation data were calculated using the 2-ΔΔCt method.

### 4.5. Western Blot

Total protein was extracted after lysis, and the protein concentration was detected using a BCA kit (Abcam, Cambridge, UK). The protein samples were denatured at 100 °C. After electrophoresis, membrane transfer, sealing, incubation with primary and secondary antibodies, and exposure, the proteins were visualized using photography. The MyoD antibody (ABclonal, Wuhan, China) and GAPDH antibody (Bioss, Beijing, China) were used for this assay.

### 4.6. Immunofluorescence Staining

The cells were fixed and permeabilized. After 1 h of isolation with 2% ready-made goat serum, they were incubated with primary antibody MyHC (Abcam, Cambridge, UK) overnight at 4 °C. A fluorescent secondary antibody, goat anti-mouse IgG, was then added and the cells were incubated at room temperature in the dark for 1 h. After the secondary antibody was removed, DAPI dye was added, and the cells were subsequently incubated for 10 min at room temperature. An inverted fluorescence microscope was finally used to capture images.

### 4.7. Cell Proliferation Assay

The transfected myoblasts were treated with 10 μL of CCK-8 (MultiSciences, Hangzhou, China). After incubation at 37 °C for 3 h, the absorbance of each sample was detected at 450 nm using a microplate reader. Cell proliferation was also detected using an EdU assay kit (RiboBio, Guangzhou, China). Images were obtained by fluorescence microscopy.

### 4.8. Dual-Luciferase Reporter Gene Assay

The psiCHECK2-circIGF1R-Wt and psiCHECK2-circIGF1R-Mut vectors were constructed, and the primer sequences are listed in Table S3. HEK293T cells were transfected with miR-16 mimics+psiCHECK2-circIGF1R-Wt; miR-16 mimics+psiCHECK2-circIGF1R-Mut; mimics NC+psiCHECK2-circIGF1R-Wt; mimics NC+psiCHECK2-circIGF1R-Mut. After transfection for 24 h, firefly and Renilla luciferase activities were detected using the Dual-Luciferase Reporter Assay Kit (Promega, Madison, WI, USA). Finally, the ratio of Renilla to firefly luciferase was calculated.

### 4.9. RIP Assay

Magna RIP RNA-Binding Protein Immunoprecipitation Kit (Millipore, Boston, MA, USA) was used for RIP assay. The antibodies included anti-AGO2 (Boster, Wuhan, China) and anti-IgG (ABclonal, Wuhan, China). The beads were then incubated with cell lysate to immune-precipitate the RNAs bound to the beads. Finally, the levels of circIGF1R and miR-16 were detected using qRT-PCR.

### 4.10. Prediction of CircIGF1R Translation Function

The CPAT (http://lilab.research.bcm.edu/ (accessed on 20 October 2022) ) and ORF Finder (https://www.ncbi.nlm.nih.gov/orffinder/ (accessed on 1 October 2022)) online websites were used to predict the open reading frame (ORF) of circIGF1R and its translation potential. The IRESite (http://www.iresite.org/ (accessed on 10 October 2022)) was used to predict whether circIGF1R has a ribosome entry site (IRES), and the SRAMP online website (http://www.cuilab.cn/sramp (accessed on 16 October 2022)) was used to predict circIGF1R m6A methylation sites.

### 4.11. Statistical Analysis

All data are shown as mean ± SEM. Statistical analyses were performed using SPSS 22.0. Data differences between the two groups were analyzed using the Student’s *t*-test, and the one-way analysis of variance was used to compare three or more groups. * *p* < 0.05, and ** *p* < 0.01.

## 5. Conclusions

In conclusion, the present study screened 362 DEcircRNAs that may regulate skeletal muscle development. In addition, we demonstrated that circIGF1R can promote myoblast differentiation of porcine SMSCs, and a new mechanism for regulating porcine myogenesis, circIGF1R-miR-16, has been identified. These results provide stronger evidence that circRNAs involved in porcine muscle development acting as a miRNA sponge. The results of this study are useful for improving pork yield and quality.

## Data Availability

The sequencing data were deposited in the Sequence Read Archive with the accession number SRP068558 (https://trace.ncbi.nlm.nih.gov/Traces/sra_sub/sub.cgi?login=pda (accessed on 10 October 2022)).

## Supplementary Materials

The following supporting information can be downloaded at: https://www.mdpi.com/article/10.3390/ijms24043779/s1.
