# Supplementary material for: CircRNA Profiling of Skeletal Muscle in Two Pig Breeds Reveals CircIGF1R Regulates Myoblast Differentiation via miR-16
Source: Int J Mol Sci. 2023 Feb 14;24(4):3779. doi: 10.3390/ijms24043779 (PMC9965117; doi:10.3390/ijms24043779)
Supplement: Supplementary file 1 [file ijms-24-03779-s001.zip › Table S1.pdf]

Table S1 Primer sequences of mRNAs

Primer names	Species	Primers sequences (5′→3′)	Product size/bp
CircIGF1R convergent primers	pig/mouse	F: GACATCCGCAACGACTAC<br>R: GAACAGCAGCAGGTACTC	153
CircIGF1R divergent primers	pig/mouse	F: GTGTGAGAAGACGACCAT<br>R: GGAGATGAGCAGGATGTG	169
MyoG	mouse	F: GTGAATGCAACTCCCACAGC<br>R: CCACGATGGACGTAAGGGAG	138
MyoD	mouse	F: GGAAGAATGCTGCGGTGTCG<br>R: CATCCCTCCGGCCCATAAGC	293
MyHC	mouse	F: ACTTGTGGTGTCGGTCACTC<br>R: CTGAAAATCAGCCGCACGTC	219
Myf5	mouse	F: AGTTCGGGGACGAGTTTGAG<br>R: TCAAACGCCTGGTTGACCTT	232
MyoG	Pig	F: CAGCCCAGCGAGGGAATTTA<br>R: GGTCAGGGCACTCATGTCTC	205
MyoD	Pig	F: CTCCTGGCACCCACTTTTCC<br>R: TTCCAACACCTGACTCGCCC	266
MyHC	Pig	F: ACGCACCCTCACTTTGTACGC<br>R: CTCTGCCGGAAGTCCCCATAG	170
Myf5	pig	F: CGGATCACGTCTACAGAGCC<br>R: GCAGGAGTGATCATCGGGAG	157
18S rRNA	pig	F: CCCACGGAATCGAGAAAGAG<br>R: TTGACGGAAGGGCACCA	122
β-actin	mouse	F: CCAGGTCATCACCATCGG<br>R: CCGTGTTGGCGTAGAGGT	158
