# Supplementary material for: CircRNA Profiling of Skeletal Muscle in Two Pig Breeds Reveals CircIGF1R Regulates Myoblast Differentiation via miR-16
Source: Int J Mol Sci. 2023 Feb 14;24(4):3779. doi: 10.3390/ijms24043779 (PMC9965117; doi:10.3390/ijms24043779)
Supplement: Supplementary file 1 [file ijms-24-03779-s001.zip › Table S2.pdf]

Table S2　Primer sequences of miRNAs

Primers names	Primers sequences (5′→3′)
*U*6	F: CTCGCTTCGGCAGCACA
	R: AACGCTTCACGAATTTGCGT
miR-16	RT: GTCGTATCCAGTGCAGGGTCCGAGGTATTC GCACTGGATACGACCGCCAA
	F: CGCGTAGCAGCACGTAAATA
	R: AGTGCAGGGTCCGAGGTATT
