# Supplementary material for: CircRNA Profiling of Skeletal Muscle in Two Pig Breeds Reveals CircIGF1R Regulates Myoblast Differentiation via miR-16
Source: Int J Mol Sci. 2023 Feb 14;24(4):3779. doi: 10.3390/ijms24043779 (PMC9965117; doi:10.3390/ijms24043779)
Supplement: Supplementary file 1 [file ijms-24-03779-s001.zip › Table S3.pdf]

Table S3 The primer sequences of circIGFR in dual-luciferase reporter gene experiments

Primers names	Primers sequences (5’→3’)
psiCHECK2-circIGF1R-Wt	sense：TCGAGAGCTCACGGTCATCACCGAGTACCTGCTGCTGTTCC GTGTGGCTGGCCTTGAGAGCGC<br>Anti-senes：GGCCGCGCTCTCAAGGCCAGCCACACGGAACAGCAG CAGGTACTCGGTGATGACCGTGAGCTC
psiCHECK2-circIGF1R-Mut	sense：TCGAGAGCTCACGGTCATCACCGAGTACCTCGACGAGTTCC GTGTGGCTGGCCTTGAGAGCGC<br>Anti-senes：GGCCGCGCTCTCAAGGCCAGCCACACGGAACTCGTCG AGGTACTCGGTGATGACCGTGAGCTC
