# Supplementary figures and images for: CircRNA Profiling of Skeletal Muscle in Two Pig Breeds Reveals CircIGF1R Regulates Myoblast Differentiation via miR-16
Source: Int J Mol Sci. 2023 Feb 14;24(4):3779. doi: 10.3390/ijms24043779 (PMC9965117; doi:10.3390/ijms24043779)

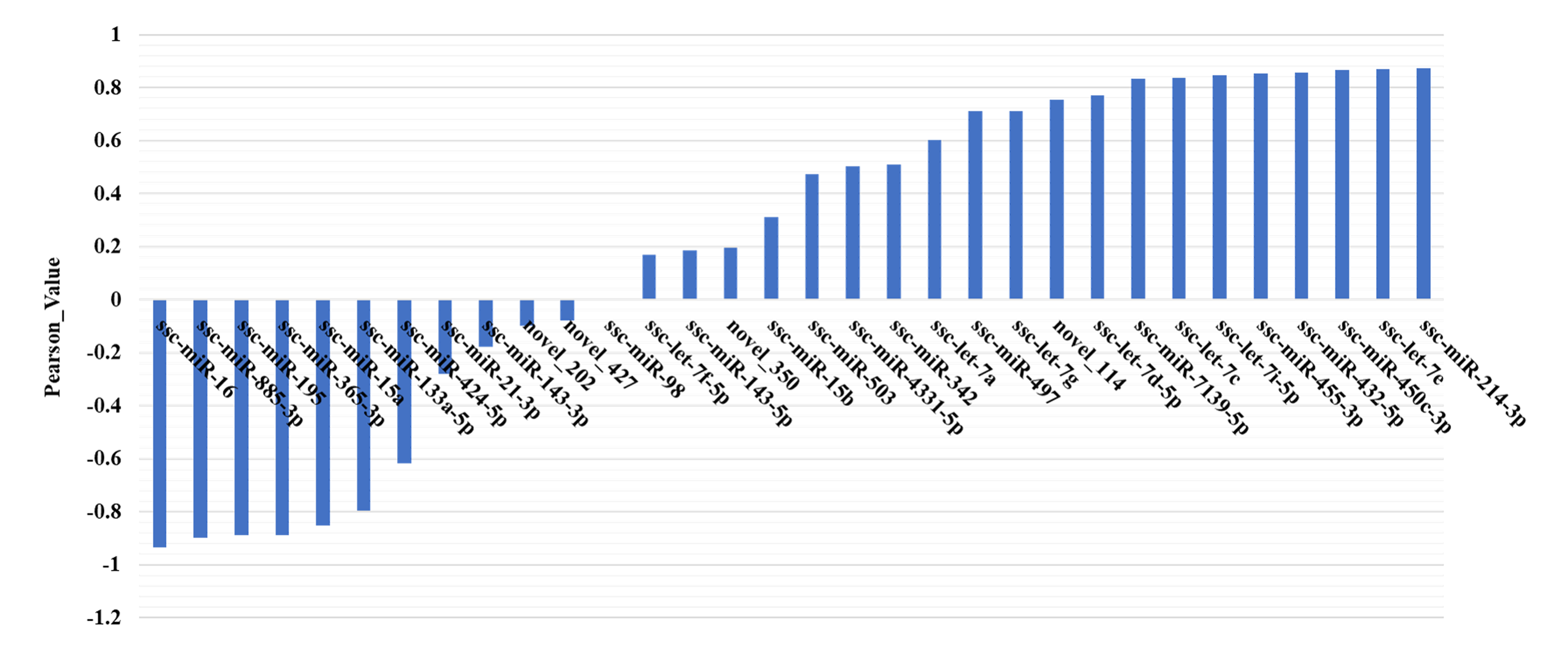

Supplement: Supplementary file 1 [file ijms-24-03779-s001.zip › Figure S1.tif]

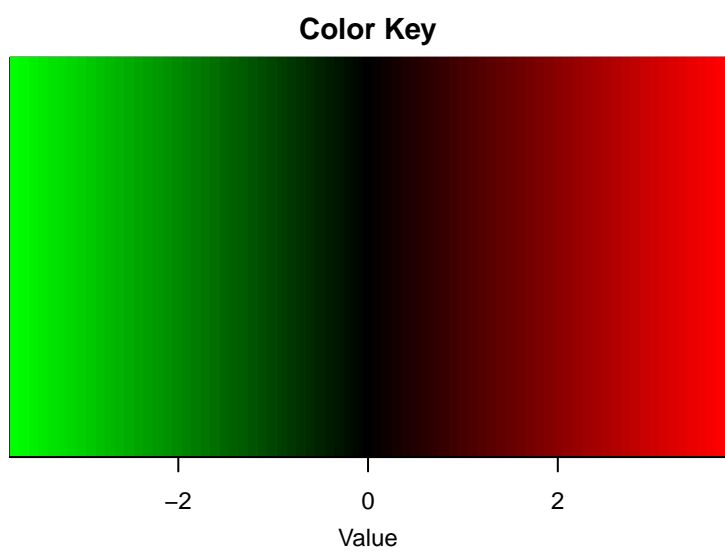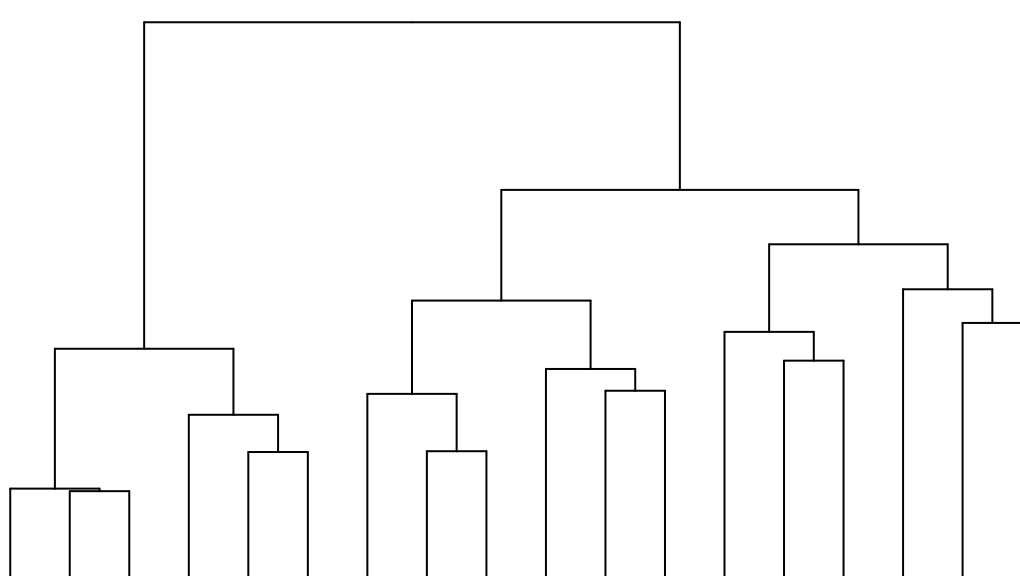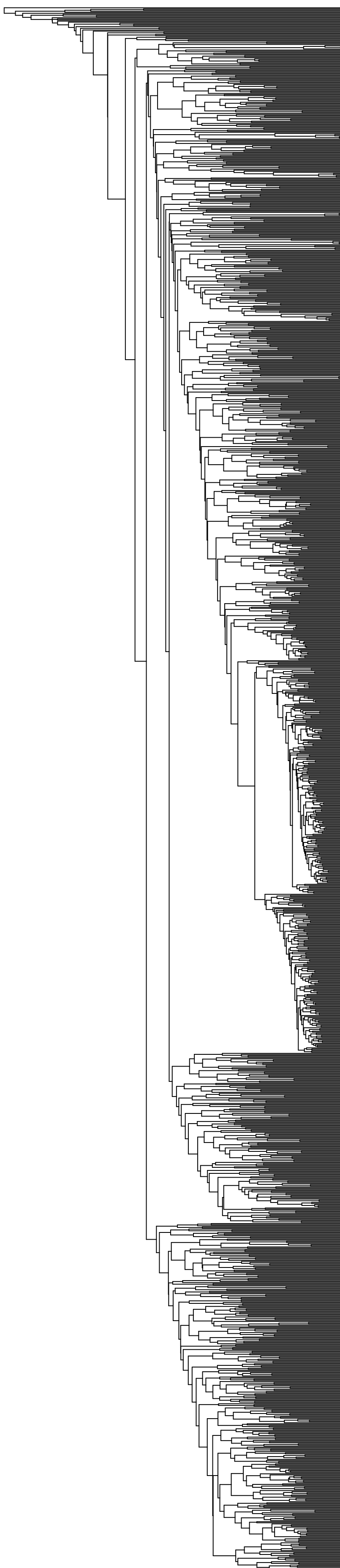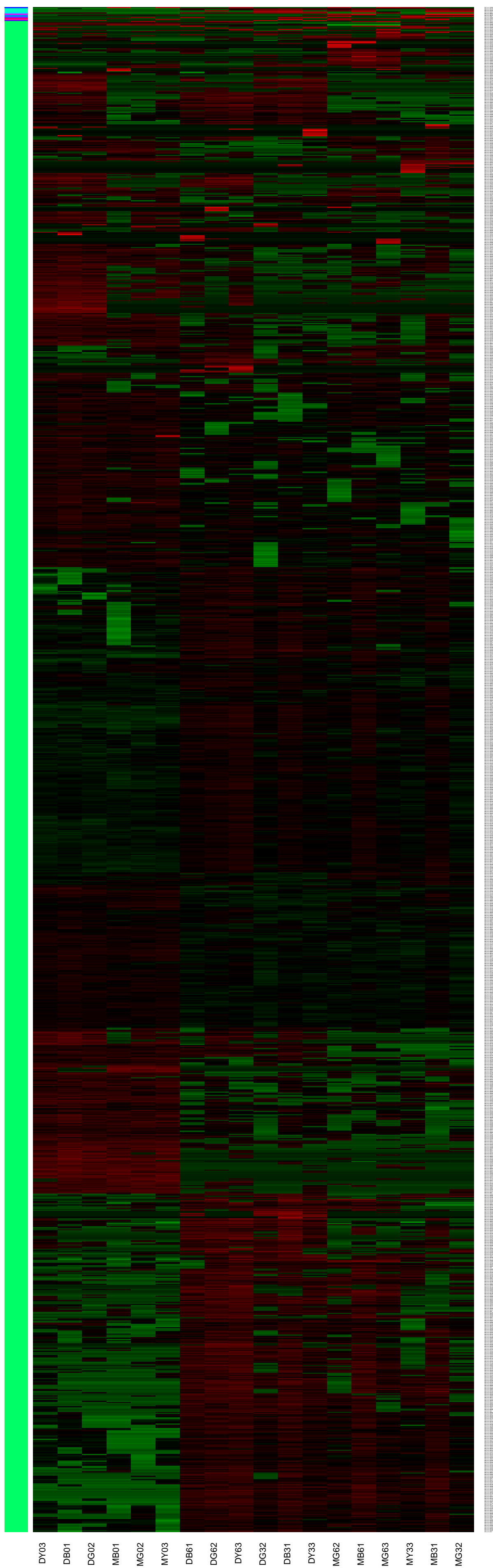

|      |      |      |      |      |      |      |      |      |      |      |      |      |      |      |      |      |      |
|------|------|------|------|------|------|------|------|------|------|------|------|------|------|------|------|------|------|
| DY03 | DB01 | DG02 | MB01 | MG02 | MY03 | DB61 | DG62 | DY63 | DG32 | DB31 | DY33 | MG62 | MB61 | MG63 | MY33 | MB31 | MG32 |
|------|------|------|------|------|------|------|------|------|------|------|------|------|------|------|------|------|------|

Supplement: Supplementary file 1 [file ijms-24-03779-s001.zip › Figure S2.pdf]
